# The neuropsychiatric approach to diagnosing and treating adults with ADHD

**DOI:** 10.1192/j.eurpsy.2025.100

**Published:** 2025-08-26

**Authors:** S. Lodhi

**Affiliations:** Department of Psychiatry, Harvard Medical School, Boston, United States

## Abstract

**Background:**

Attention-deficit/hyperactivity disorder (ADHD) in adults often presents with nonspecific symptoms that can be obscured by years of compensatory mechanisms and comorbid conditions. Traditional diagnostic approaches may fall short in capturing the nuanced presentation of ADHD in previously undiagnosed adults, leading to potential misdiagnosis or delayed treatment. This talk presents a comprehensive clinical framework for evaluating adult ADHD that integrates structured assessment protocols with thorough clinical investigation.

**Methods:**

Our diagnostic model employs a multi-stage evaluation process that includes: (1) comprehensive pre-interview rating scales, (2) in-depth clinical interviews focusing on lifetime symptom presentation and functional impact, (3) systematic gathering of collateral information, and (4) administration of validated assessment instruments with embedded validity scales. The evaluation protocol emphasizes the assessment of executive function, emotional regulation, and adaptive behaviors developed throughout adulthood.

**Discussion:**

This approach represents a significant advancement in adult ADHD diagnosis by incorporating validated assessment tools with thorough clinical investigation. The model’s emphasis on lifetime symptom patterns, adaptive behaviors, and compensatory mechanisms provides crucial insights into how ADHD manifests in adults who have developed various coping strategies throughout their lives. Furthermore, the integration of multiple data sources with patient insights facilitates better treatment engagement and outcomes.

**Conclusion:**

The proposed comprehensive evaluation framework offers a more sophisticated and nuanced approach to diagnosing ADHD in previously undiagnosed adults. By combining validated assessment instruments with thorough clinical investigation and collateral information, this model enhances diagnostic accuracy and treatment planning. Our findings suggest that this approach not only improves diagnostic precision but also provides valuable insights into the developmental trajectory of ADHD, leading to more personalized and effective treatment strategies.

**Keywords:**

Adult ADHD, clinical evaluation, executive function, compensatory mechanisms, diagnostic precision

**Disclosure of Interest:**

None Declared

